# Lipid Metabolism Reprogramming and Trastuzumab Resistance in Breast Cancer Cell Lines Overexpressing the ERBB2 Membrane Receptor

**DOI:** 10.3390/membranes13060540

**Published:** 2023-05-23

**Authors:** Katia Cortese, Marco Ponassi, Aldo Profumo, Gabriela Coronel Vargas, Erika Iervasi, Maria Cristina Gagliani, Grazia Bellese, Sara Tavella, Patrizio Castagnola

**Affiliations:** 1 DIMES, Department of Experimental Medicine, Cellular Electron Microscopy Lab, Università di Genova, Via Antonio de Toni 14, 16132 Genova, Italy; gagliani@unige.it (M.C.G.); bgrazia@unige.it (G.B.); 2 IRCCS Ospedale Policlinico San Martino, Largo Rosanna Benzi 10, 16132 Genova, Italy; marco.ponassi@hsanmartino.it (M.P.); aldo.profumo@hsanmartino.it (A.P.); gabrielafernanda.coronelvargas@edu.unige.it (G.C.V.); erika.iervasi@hsanmartino.it (E.I.); or sara.tavella@unige.it (S.T.); 3 DIMES, Department of Experimental Medicine, Cellular Oncology Unit, Università di Genova, Largo Rosanna Benzi 10, 16132 Genova, Italy

**Keywords:** trastuzumab, trastuzumab resistance, HER2, breast cancer, proteomic analysis, metabolic reprogramming, subcellular organelles, membrane-associated pathways

## Abstract

Trastuzumab (Tz), an antibody targeting ERBB2, has significantly improved the prognosis for breast cancer (BCa) patients with overexpression of the ERBB2 receptor. However, Tz resistance poses a challenge to patient outcomes. Numerous mechanisms have been suggested to contribute to Tz resistance, and this study aimed to uncover shared mechanisms in in vitro models of acquired BCa Tz resistance. Three widely used ERBB2+ BCa cell lines, adapted to grow in Tz, were examined. Despite investigating potential changes in phenotype, proliferation, and ERBB2 membrane expression in these Tz-resistant (Tz-R) cell lines compared to wild-type (wt) cells, no common alterations were discovered. Instead, high-resolution mass spectrometry analysis revealed a shared set of differentially expressed proteins (DEPs) in Tz-R versus wt cells. Bioinformatic analysis demonstrated that all three Tz-R cell models exhibited modulation of proteins associated with lipid metabolism, organophosphate biosynthesis, and macromolecule methylation. Ultrastructural examination corroborated the presence of altered lipid droplets in resistant cells. These findings strongly support the notion that intricate metabolic adaptations, including lipid metabolism, protein phosphorylation, and potentially chromatin remodeling, may contribute to Tz resistance. The detection of 10 common DEPs across all three Tz-resistant cell lines offers promising avenues for future therapeutic interventions, providing potential targets to overcome Tz resistance and potentially improve patient outcomes in ERBB2+ breast cancer.

## 1. Introduction

Tumors overexpressing the ERBB2 receptor, a member of the EGFR/ERBB1 receptor family, are about 25% of all breast cancers (BCa) and are associated with poor prognosis, since the disease has an aggressive character and a high propensity to metastasize [1,2]. Overexpression of ERBB2 causes overactivation of the MAPK/ERK and PI3 kinase/AKT pathways, which promote cell proliferation, differentiation, migration, and angiogenesis [3,4,5]. The introduction of the recombinant and humanized monoclonal antibody named trastuzumab (Tz), directed against the extracellular domain IV of ERBB2 in the therapy regimen, significantly improved the patient’s overall survival and disease-free survival [6,7]. Recently, novel Tz-based therapeutics have been developed, further reinforcing its crucial role in the management of ERBB2 BCa patients [8]. Tz impairs the survival of ERBB2-overexpressing cells and promotes antibody-dependent cell-mediated cytotoxicity [9,10]. Several mechanisms have been proposed to contribute to the Tz-induced inhibition of survival of ERBB2-overexpressing cells in vitro [11,12], which may partly dependent on the particular cell model used. Inhibition of ERBB2 growth signaling through the PI3K/AKT pathway [13] and accumulation of active CDKN1B in the nucleus, leading to Cdk2 inactivation and arrest in the G0/G1 phase of the cell cycle, is the most commonly acknowledged one [14]. Despite Tz effectiveness, about 35% of ERBB2+ breast cancer patients are de novo resistant to Tz, and among those that are responsive, about 70% will eventually become resistant within one year of treatment [15]. Several molecular mechanisms have been proposed as responsible for Tz resistance [16]. These can be summarized into two broad categories: intrinsic to the cancer cells and extrinsic [e.g., host immune system-related]. Among the former, the following may be included: (1) ERBB2 truncation, (2) impaired binding of Tz to HER2, (3) activation of compensatory or alternative signaling pathways, (4) defects in apoptosis and cell cycle control, (5) increased ability to generate cancer stem cells, (6) vascular mimicry and hypoxia, and (7) metabolic adaptation. Although extrinsic mechanisms may be of major relevance in naïve patients, it is conceivable that in patients receiving chemotherapy in the neoadjuvant setting, likely experiencing with time an immune-suppression state, intrinsic mechanisms of resistance may play a relevant role. We believe that despite limitations, an in vitro study may provide some insight into these intrinsic mechanisms. Using an unbiased approach, we aimed to reveal whether a cellular process could be associated with the development of acquired resistance to Tz in ERBB2+ BCa cell lines, with differences in receptor status [17] and sensitivity to Tz [18]. In particular, we used BT474, MDA-MB-361 and SKBR-3 wt and Tz-R cells that we adapted to grow in a Tz concentration about tenfold higher than therapeutic serum levels [19]. We investigated cell morphology, cell cycle, ERBB2 expression, and proteomic profile to identify possible molecular mechanisms at the bases of acquired Tz resistance shared by the three cell lines. Our results showed that proteins involved in metabolic reprogramming were differentially expressed by all the cell lines in the two conditions, which suggests that, at least in vitro, this process may contribute significantly to the development of cell-intrinsic Tz resistance.

## 2. Materials and Methods

### 2.1. Cell Lines and Cell Culture Reagents

The BC cell lines BT474, MDA-MD-361 and SKBR-3 were obtained from Banca Biologica and Cell Factory in IRCCS Ospedale Policlinico San Martino affiliated to the European Culture Collection’s Organization. Culture media for routine cell expansion was DMEM high glucose supplemented with 1% glutamine, penicillin, streptomycin, and 10% heat-inactivated fetal bovine serum for BT474 and SKBR-3 or 20% for MDA-MD-361 (Euroclone S.p.A, Pero, Italy). To obtain Tz-R cells, each cell line was cultured and expanded in medium containing Tz at the initial concentration of 10 µg/mL that was progressively increased over about 6 months, to the final concentration of 160, 250, and 200 µg/mL for BT474, SKBR-3, and MDA-MD-361, respectively. Cells were routinely cultured in the Tz-containing medium for over a year before analysis. Wt and Tz-Res cells were cultured in parallel to minimize differences in passage number. Tz was kindly provided by the pharmacy (UFA-Unità Farmaci Antiblastici) of the IRCCS Ospedale Policlinico San Martino.

### 2.2. Flow Cytometry [FCM] Analysis and Antibodies

To determine cell number, 50,000 BT747 wt and Tz-R cells, 75,000 MDA-MB-361 wt and Tz-R cells, and 35,000 SKBR-3 wt and Tz-R cells were seeded per well in a Corning 12 well plate (22.1 mm in diameter) (Corning, Corning, NY, USA) and the counts were performed after trypsinization by FCM. ERBB2 surface-expression levels were investigated using Tz. A goat anti-human Alexa 647-conjugated antibody was used to detect Tz (Thermo Fisher Scientific, Waltham, MA, USA). Cells were analyzed and counted using a CyFlow ML flow cytometer performing True Volumetric Absolute Cell Counting [TVAC] (Sysmex-Partec Inc., Lincolnshire, IL, USA) or a Cytoflex flow cytometer (Beckman Coulter, IN, USA).

### 2.3. Cell Imaging

Phase-contrast images of sub-confluent cell cultures were taken using an inverted Nexcope NIB620 microscope equipped with a Peltier-cooled FL-2 CCD camera (Bresser GmbH, Rhede, Germany).

### 2.4. Sample Preparation, Mass Spectrometer Setup and Protein Identification

Cell lines were cultured and treated in 75 cm^2^ flasks. When treatment was completed, cells were washed twice in 1× PBS and then harvested by scraping. After centrifugation, cell pellets were resuspended in RIPA buffer [protease and phosphatase inhibitors and 1 mM DTT were added] and then frozen at −80 °C. From this point, all the procedures were performed at 4 °C or in ice. Once thawed, cell lysates were resuspended every 15 min for four times and then sonicated for 30 s with pulse [output approximately 10 watts]. Then, samples were centrifugated at 13,850 rcf for 10 min. Supernatants were collected and an equal volume of 20% SDS-6% DTT was added. The samples were incubated at 95 °C for 5 min. Then, five volumes of MATF (Methanol, Acetone and Tributhyl phosphate, 1:12:1) were added and samples were incubated for 1 h on a rotating wheel. The total proteins were finally precipitated by centrifugation at 12,000 rcf for 15 min, the supernatants were eliminated and the protein pellets were dried for about 30 min at RT in a Savant SpeedVac apparatus (Thermo Fisher Scientific). Each dried pellet was resuspended with 250 µL of 5% SDS in 50 mM Ammonium Bicarbonate [AMBIC]. After a brief centrifugation, 2 µL of each sample were processed to determine the protein concentration using the QuantiPro BCA Assay Kit (Sigma-Aldrich, St. Louis, Mo, USA). Afterward, 20 mM DTT was added to 50 µg of total proteins for each sample and the samples were incubated at 95 °C for 10 min. Once the samples were cooled to RT, Iodoacetamide was added to a final concentration of 40 mM, and then the reaction mixtures were incubated for 30 min in the dark. Finally, orthophosphoric acid was added to a final concentration of 1.2%. The reduced and alkylated protein samples were loaded onto S-Trap mini columns, washed and then trypsin-digested as suggested by the manufacturer (Protifi, Farmingdale, NY, USA). The eluted mixtures were dried at RT.

To perform mass spectrometry analysis, two biological replicates were performed for each cell line and Tz resistance status, and both were analyzed twice by LC/MS-MS to obtain four datasets (see Table S1). To perform mass spectrometry analysis, the desiccated tryptic digests were resuspended with 0.2% formic acid in water and analyzed by nano-UHPLC-MS/MS using an Ultimate 3000 chromatography system, equipped with a PepMap RSLC C18 EASY spray column [75 µm × 50 cm, 2 µm particle size] (Thermo Fisher Scientific) at a flow rate of 250 nL/min and a temperature of 60 °C. The following mobile phase composition has been used: (A) 0.1% *v*/*v* formic acid in water and (B) 80% ACN, 20% H_2_O and 0.08% *v*/*v* formic acid. A 105 min gradient was selected: 0.0–3.0 min isocratic 2% B; 3.0–7.0 min 7% B; 7.0–65.0 min 30% B; 65.0–78.0 min 45% B; 78.0–83.0 min 80% B; 83.0–85.0 isocratic 80% B; 85.0–85.1 2% B and finally 85.1–105.0 isocratic 2% B. After separation, the flow was directly sent to an Easyspray source connected to a Q Exactive™ Plus Hybrid Quadrupole-Orbitrap™ mass spectrometer (Thermo Fisher Scientific). The data were acquired in data-dependent mode, alternating between MS and MS/MS scans. The software Xcalibur (version 4.1, Thermo Fisher Scientific) was used for operating the UHPLC/HR-MS. MS scans were acquired at a resolution of 70,000 between 200 and 2000 *m*/*z*, with an automatic gain control [AGC] target of 3.0 × 10^6^ and a maximum injection time [maxIT] of 100 ms. MS/MS spectra were acquired at a resolution of 17,500 with an AGC target of 1.0 × 10^5^ and a maxIT of 50 ms. A quadrupole isolation window of 2.0 *m*/*z* was used, and HCD was performed using 30 normalized collision energy [NCE]. The mass spectrometry proteomics data were deposited into the ProteomeXchange Consortium via the PRIDE [20] partner repository with the dataset identifier PXD037657. Data from mass spectrometer in *.raw format was processed with ProteomeDiscoverer^®^ software version 2.4.1.15 (Thermo Fisher Scientific) for PMSs identification in MS/MS spectra, PMSs and protein quantification and for differential proteomics as explained in the “Data Processing Protocol” paragraph of the PXD037657 project. Peptide abundances were normalized based on total peptide amount and scaled on all averages. Proteins were quantified using IMP-apQuant node by summed abundances, pairwise ratio-based and t-test background-based. Significative differentially expressed proteins [DEPs] between wt and Tz-resistant ERBB2+ cell lines were identified by averaging protein abundances from two biological samples and two technical replicates for each cell line and Tz resistance status (Table S1) and using a Log_2_ Fold change > 2 and a *p* < 0.05.

### 2.5. Electron Microscopy

Wild type and Tz-res cell lines BT474, MDA-MD-361 and SKBR-3 were seeded overnight on glass chamber slides (Lab-Tek 177380, Nalge Nunc int., Rochester, NY, USA). Cells were washed out in 0.1M cacodylate buffer and fixed in 0.1M cacodylate buffer containing 2.5% glutaraldehyde (Electron Microscopy Science, Hatfield, PA, USA), for 1 h at RT. Samples were postfixed in osmium tetroxide for 2 h and 1% uranyl acetate for 1 h. Cells were next dehydrated through a graded ethanol series and flat-embedded in resin (Poly-Bed; Polysciences, Inc., Warrington, PA, USA) for 24 h at 60 °C. Ultrathin sections [50 nm] were cut and stained with 5% uranyl acetate in 50% ethanol and observed with a Hitachi 7800 electron microscope (Hitachi, Tokyo, Japan) [21]. Digital images were taken with a Megaview III camera and Radius 2.0 software (EMSIS, Muenster, Germany). Analysis of number and size of morphologically identified lipid droplets (LDs) was conducted in 10 randomly chosen cells for each condition. The diameter of each organelle was measured with the line tool of Radius 2.0 software (EMSIS, Muenster, Germany) and plotted as bar plots ± SEM.

### 2.6. Statistical Analyses

Analyses was performed using Prism (GraphPad Software, La Jolla, CA, USA). We used the multiple unpaired t-test to evaluate cell proliferation differences. To measure cell survival, we used the unpaired t-test with Welch’s correction. A Brown-Forsythe and Welch ANOVA test with a Dunnett’s T3 multiple comparisons test was used to evaluate ERBB2 expression levels by FCM analysis. A t-test was performed to evaluate lipid droplet (LD) size and number. Mean differences were considered statistically significant at *p* < 0.05.

## 3. Results

### 3.1. Generation and Characterization of ERBB2+ Tz-R Cell Lines

**Figure 1:**
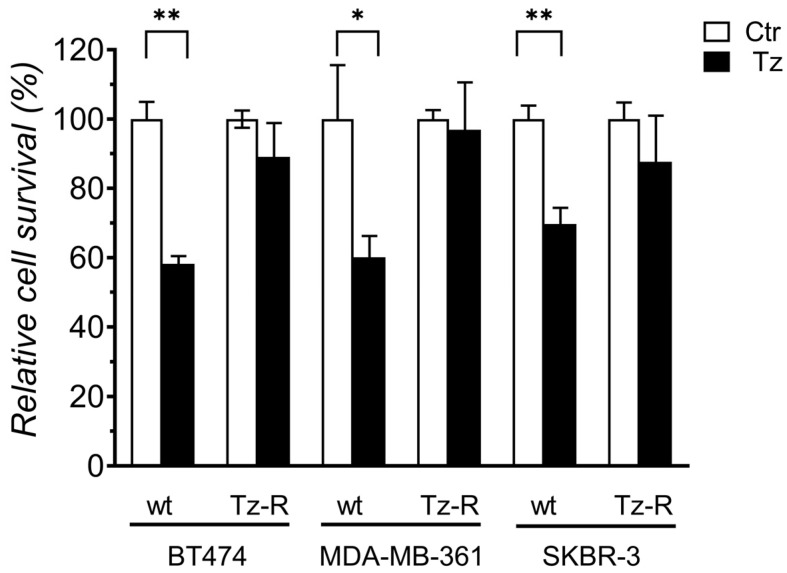
Cell survival analysis of ERBB2+ BCa cell lines treated with 10 µg/mL Tz compared with controls [Ctr]. *p*-values between each control and treated sample for each cell line are shown: *p* < 0.05 [*], *p* < 0.01 [**].

To obtain Tz-R cells, BT474, MDA-MD-36, and SKBR-3 were cultured and expanded in medium containing Tz at the initial concentration of 10 µg/mL progressively increased over about 6 months, to the final concentration of 160, 200, and 250 µg/mL for BT474, MDA-MD-361, and SKBR-3, respectively. Cells were routinely cultured in the Tz-containing medium for a further year before analysis. BT474, MDA-MB-361 and SKBR-3 Tz-R cells showed no statistically significant difference in survival rate after 72 h of treatment with 100 µg/mL Tz vs. controls, while wt cells showed a statistically significant reduction corresponding to 58.2, 60.1, and 69.7%, respectively, vs. controls (Figure 1).

**Figure 2:**
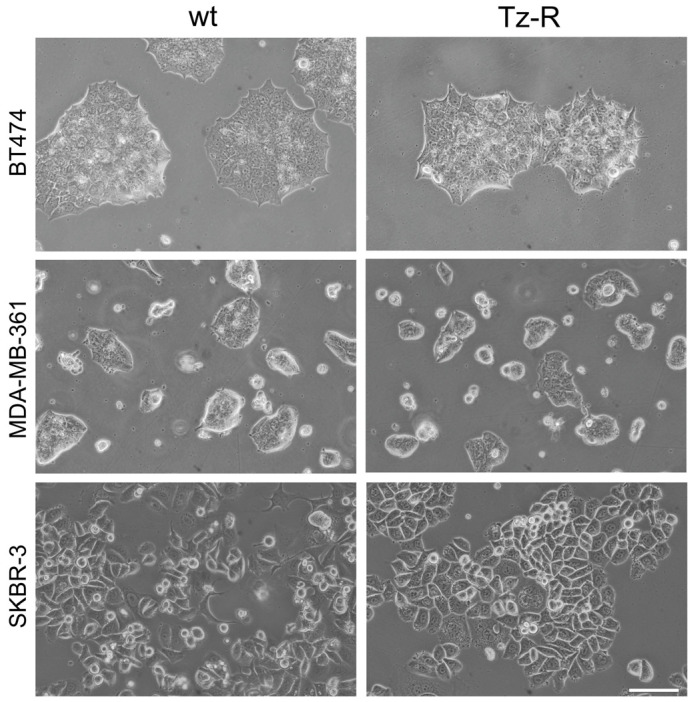
Phase-contrast images of ERBB2+ BCa wt and Tz-R cell lines used in this study. Scale bar = 200 µm.

Microscopic analysis in bright field and phase contrast showed that SKBR-3 Tz-R with respect to wt cells displayed a more homogeneous and typical epithelial phenotype with a large portion of the plasma-membrane involved in extended intercellular contacts. No major differences were observed instead between BT474 and MDA-MB-361 wt and Tz-R cells (Figure 2).

**Figure 3:**
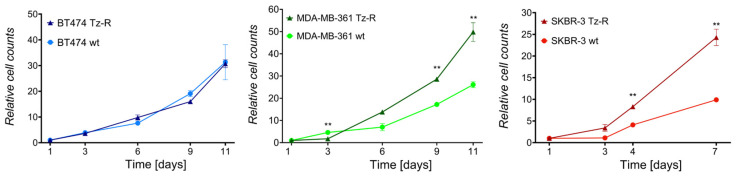
Growth curves of ERBB2+ BCa wt and Tz-R cell lines used in this study. Cell counts at each time point are reported as fold changes with respect to those counted 1 day after seeding for each culture. Mean values and SD [indicated as vertical bars] are reported. Significant adjusted *p*-values are shown: *p* < 0.01 [**].

As a previous report showed a reduced growth rate in Tz-R cell lines derived from gastric cancer [22], we investigated whether reduced proliferation was a character shared by all three of our Tz-R cell lines. In particular, we performed cell-growth curves by counting cells recovered from cultures at several time points after seeding. This analysis showed, instead, that a higher number of cells was significantly recovered from SKBR-3 Tz-R and MDA-MB-361 Tz-R in at the last two time points of the analysis than in wt cultures, while no difference was observed between BT474 Tz-R and wt (Figure 3).

**Figure 4:**
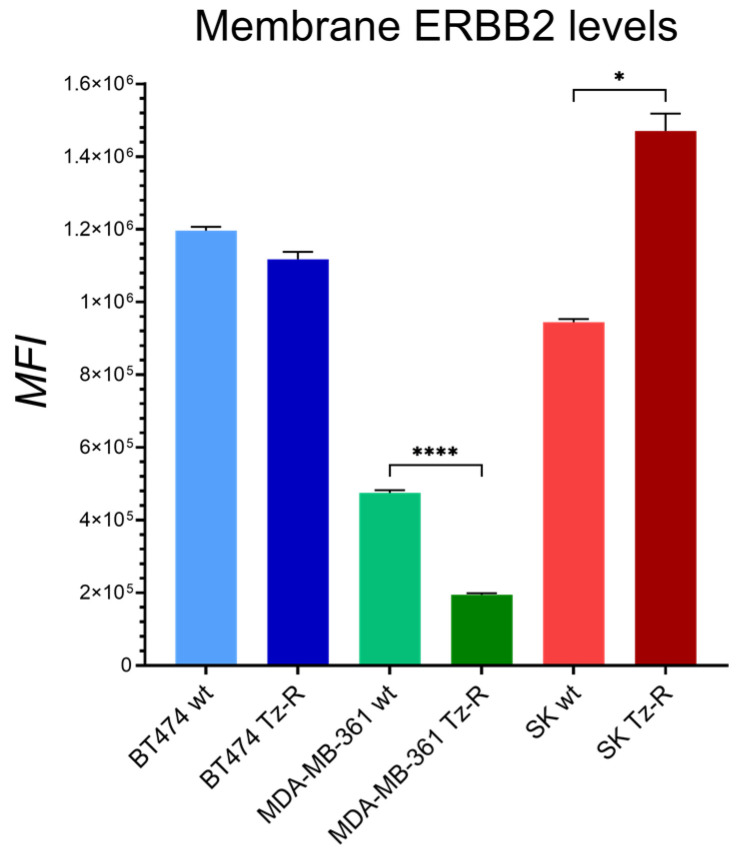
Plasma membrane ERBB2 median fluorescence intensity [MFI] expression levels in ERBB2+ BCa cell lines. All cell lines were first incubated with Tz and then with an anti-human Alexa 647 antibody. Only significant adjusted *p*-values between each wt and Tz-R cells belonging to the same cell line are shown: *p* < 0.05 [*], *p* < 0.0001 [****]. A complete list of the statistical analysis results is provided in Table S2.

As downregulation of ERBB2 after Tz therapy has been observed in vitro and in vivo, and this may promote resistance toward Tz [16,23], we evaluated whether this mechanism was present in all our Tz-R cell models at the level of the plasma membrane [PM] where ERBB2 plays its main role. FCM analysis with the Tz antibody used to detect ERBB2 revealed that MDA-MB-361 Tz-R displayed a robust and statistically significant decrease in the levels of ERBB2 exposed on the PM. On the contrary, SKBR-3 Tz-R displayed a significant increase of surface-exposed ERBB2, while BT474 Tz-R cells showed only minor non-significant changes in ERBB2 PM levels with respect to wt cells (Figure 4). Full statistical significance results of this FCM analysis are provided in Table S2.

### 3.2. Proteomic Analysis of ERBB2+ and Tz-R Cell Lines Reveals Deregulation of Lipid Metabolism, Organophosphate Biosynthetic Process, and Macromolecule Methylation

**Figure 5:**
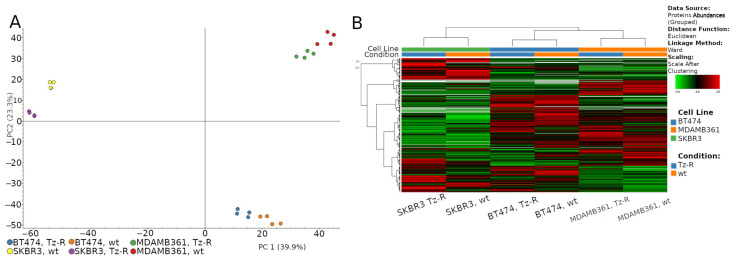
(**A**) Two-dimensional scatter plot of the principal component analysis of proteins expressed by wt and Tz-R ERBB2+ BCa cell lines used in this study. (**B**) Unsupervised hierarchical-clustered heatmap of identified proteins. The amount of each protein in individual samples is represented by the color scheme in which red and green indicate high and low expression of proteins, respectively. Proteins are clustered into three groups according to their expression value, which correspond to the three main cell lines.

To gain insights into the protein associated with the acquisition of Tz resistance, we used a proteomic and unbiased approach relying on high-resolution mass spectrometry on whole lysate of BT474, MDA-MB-361, and SKBR-3 wt and their counterpart Tz-R cells. This analysis was performed using two biological replicates and each replicate was run two times. Similar protein abundance was detected in each sample (Supplementary File S1). In particular, a range of 3141–3271 proteins with high FDR were identified in the three wt samples, while 3021–3056 were identified in the three Tz-R samples. To reveal differences or similarities between the two conditions, wt and TZ-R, we performed a principal component analysis [PCA]. The PCA analysis showed that the proteins were separated into three main groups according to the cell line and that within each of these three main groups the TZ-R samples were separated from the wt ones (Figure 5A). An unsupervised hierarchical clustering analysis showed that the modulated proteins are clustered in three well defined groups, corresponding to the three cell lines, which is in agreement with the PCA analysis (Figure 5B).

**Figure 6:**
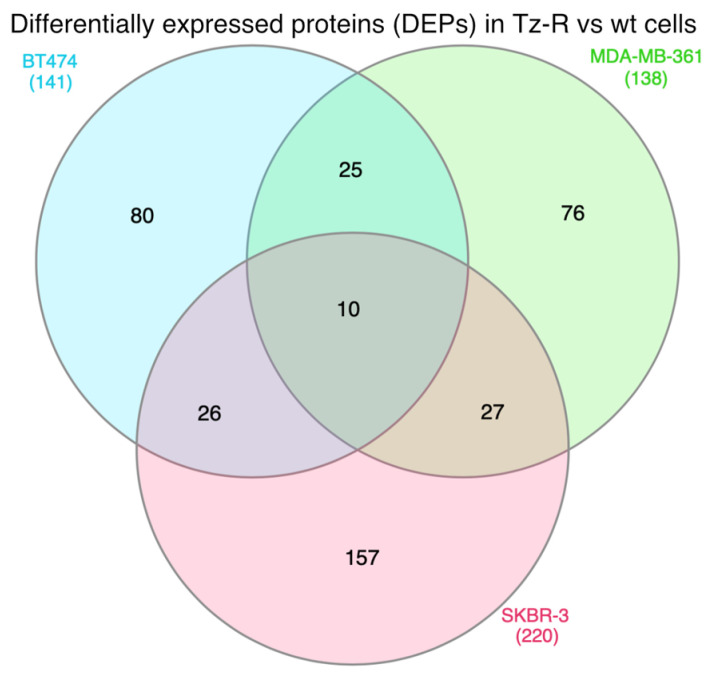
Venn diagram representing DEPs in ERBB2+ BCa Tz-R vs wt cell lines. The number of DEPs proteins identified in each cell line is reported along with the number of those shared among them.

In particular, after excluding the IgG heavy chain of the Tz present in growth medium of Tz-R cells and comparing the wt vs. TZ-R cell lines, we found 141 differentially expressed proteins [DEPs] in BT474, 138 in MDA-MB-361, and 220 in SKBR-3 (Figure 6).

**Figure 7:**
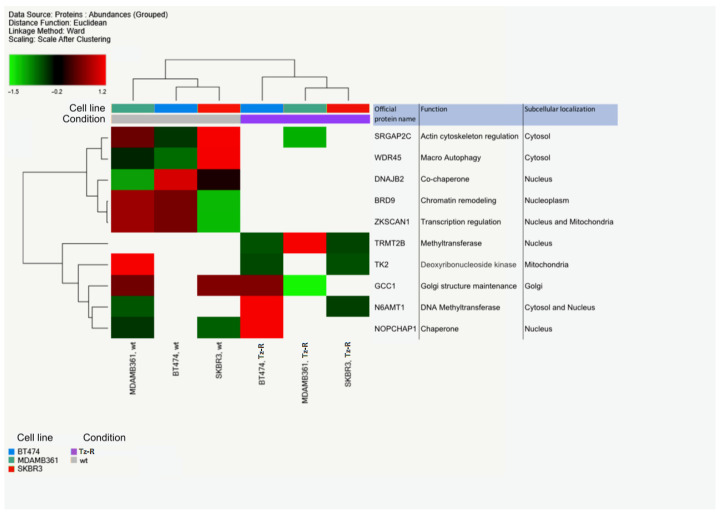
Unsupervised hierarchical-clustered heatmap of 10 DEPs that were found deregulated by all three Tz-resistant cell lines. The amount of each protein in individual samples is represented by the color scheme in which red and green indicate high and low expression of proteins, respectively. White boxes indicate an expression level below the mass spectrometry detection threshold. Proteins are clustered into two main groups according to their expression value, which correspond to the two conditions: wt and Tz-resistant cells. Official protein names, functions and subcellular localizations are indicated on the right.

A list of all these DEPs is provided (Table S3). Interestingly, we observed that the three cell lines shared 10 DEPs. Among these, we noticed that five of these DEPs were undetectable in the three Tz-R models or downregulated vs. their wt counterpart (Upper 5 protein listed in Figure 7). However, a study of the literature showed that these 10 common DEPs are associated with different cellular compartments and functions, which did not allow us to infer a common biological process deregulated in the three Tz-R cell lines (Figure 7).

As mechanisms of ERBB2-targeted resistance have been associated with changes in the expression levels of several proteins belonging to different pathways, such as ERBB receptors, RAS-MAPK, PI3K-AKT, PGR, and ER, we have analyzed the expression levels of these along with other proteins related to masking mechanisms [24,25]. In particular, protein levels from whole-cell lysates were obtained from proteomic analysis. Abundance ratios of protein expression levels in Tz-R vs wt cells found with high FDR confidence in samples from the cell lines used in the study are shown in Table S4. None of the listed markers were consistently modulated in all three Tz-R vs wt cells. Worth noting is that four proteins were found to be modulated with an abundance ratio with statistically significant adjusted *p*-values [*p* < 0.05]. These were CDK6, EGFR, MUC1, and ERBB2. CDK6 was upregulated in Tz-R BT474 while EGFR and MUC1 were upregulated in Tz-R SKBR-3 cells. ERBB2 was found to be downregulated in Tz-R MDA-MB-361.

To gain insights into biological processes deregulated in the Tz-R condition, we performed a pathway and process enrichment analysis by submitting all the DEPs found in each of the three lines to the Database for Annotation, Visualization and Integrated Discovery [DAVID] tools, and to the Metascape resource, available at https://david.ncifcrf.gov, accessed on 17 October 2022, and https://metascape.org, accessed on 27 October 2022, respectively. Using DAVID tools, we found that DEPs in our Tz-R cell models were significantly associated with specific pathways included in the KEGG collection. Interestingly, metabolic pathways were an entry shared by the three lines (Table 1).

Metascape allowed us to define more specifically that the reactome gene set ‘metabolism of lipids’, along with the ‘organophosphate biosynthetic’, and ‘macromolecule methylation’ processes, were enriched and shared identifier or terms significantly associated with DEPs across the three cell lines (Table 2).

### 3.3. Electron Microscopy Revealed Changes in Lipid Droplets Content in Tz-R Cells

**Figure 8:**
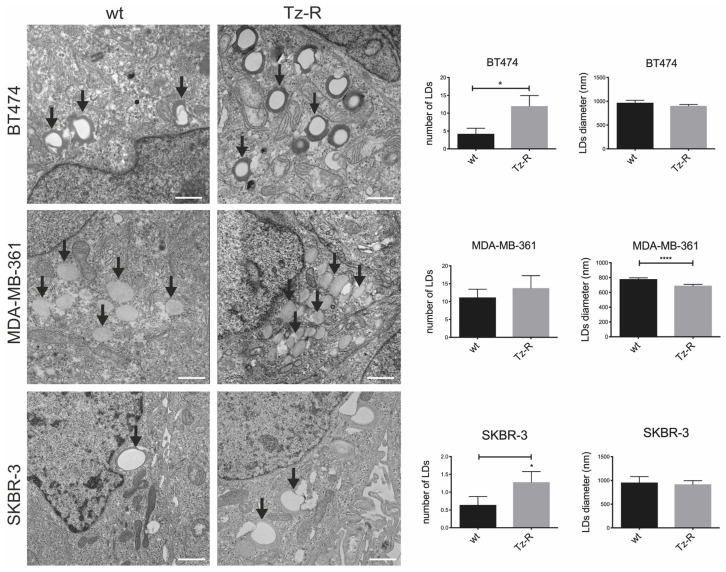
Representative TEM images of ERBB2+ BCa wt and Tz-R cell lines. Lipid droplets [LDs] were morphologically identified [black arrows]. Bar plots show the LD number and diameter measured for each experimental condition. For this analysis, 10 whole cells were scored and measured for LDs with Radius 2.0 imaging software. *p* = 0.023 [*] for SKBR-3, *p* = 0.032 [*] for BT474 [LDs number] and *p* < 0.0001 [****] for MDA-MB-361 [LDs diameter], respectively. Scale bars: 1 µm.

The results obtained from the proteomic analysis prompted us to characterize the subcellular phenotype possibly associated with the deregulation of lipid metabolism in Tz-R cells. With this aim, we performed an ultrastructural examination of lipid droplets (LDs) in BT474, MDA-MB-361, and SKBR-3 wt and their counterpart Tz-R cells. EM analysis revealed that MDA-MB-361 and SKBR-3 Tz-R cells displayed a significant increase in the number of morphologically identified LDs with comparable size, while in BT474 Tz-R cells this increase was present but not significant. However, BT474 Tz-R cells showed a significant reduction of LD size (Figure 8).

## 4. Discussion

Trastuzumab resistance remains a challenging occurrence in BCa patient management and deserves further study to be better understood. Changes elicited by Tz in the cancer cells during treatment may have a major role in treated patients with reduced immune response due to chemotherapeutics associated with Tz. in vitro studies may help in their identification. The aim of this study was to highlight a possible common mechanism at the base of trastuzumab resistance in ERBB2+ BCa cell lines. Our unbiased study points toward a metabolic reprogramming contributing to Tz resistance in ERBB2+ BCa cells derived from cell lines with different receptor status and sensitivity to Tz. In particular, we used the widely available cell lines BT474, MDA-MB-361, and SKBR-3 and adapted them to grow in medium containing a Tz concentration up to about 10 times greater than that found in the serum of treated patients.

Phase-contrast microscopy analysis showed that Tz resistance in the three cell lines does not result in common changes in cell morphology with respect to the wt counterparts. Growth curves of our cell lines showed that in our models of Tz-R cells higher proliferation was observed in MDA-MD361 and SKBR-3 Tz-R cells but not in BT474 Tz-R cells. Our results are not in agreement with those of a previous study, but the reason could be due to the different ERBB2+ tumor type studied [22]. According to proliferation analysis, ERBB2 expression analysis by FCM also showed a non-consistent modulation of its levels on the PM among our Tz-R cells. This result ruled out receptor downmodulation as a common mechanism associated with Tz resistance in our models. Similarly, proteomic data also ruled out in our models a common deregulation of proteins belonging to pathways related to ERBB2-targeted therapy resistance such as ERBB receptors, RAS-MAPK, PI3K-AKT, PGR, and ER or related to ERBB2-masking mechanisms. Proteomic analysis data, and in particular PCA, showed that each Tz-R cell line was closely related to the original wt line and that the three cell lines could be well separated from each other. In detail, we found that about 138–220 proteins were differently regulated in the Tz-R cells with respect to each wt counterpart. This indicated that by using an arbitrary Log_2_ Fold change > 2 in protein expression levels, a small set of differentially expressed proteins was associated with the Tz-resistant status. Among these DEPs, we found 10 proteins shared among the three models and hierarchical clustering clearly showed a subset of five of these DEPs that were downregulated or altogether undetected in the Tz-R cell lines. We found of particular interest that SRGAP2C and WDR45 were included in this five-DEP subset. Our observation, along with previous data showing that these two proteins are positive prognostic markers when overexpressed in cancer [26,27], suggests that the loss of functions associated with these five DEPs may play a role in promoting a more aggressive character to Tz-resistant cells. The opposite may apply to TRMT2B which was, instead, undetectable in all our ERBB2+ BCa wt cells. We also found that four proteins among these 10 DEPs were not consistently regulated in the three cell models by Tz resistance: TK2, GCC1, N6AMT1, and NOPCHAP1. Overall, our data prompt further studies to better establish the relationship of these 10 common DEPs with Tz resistance. As expected from the size of the 10-protein set shared among the Tz-R cells, the bioinformatic tools that we used could not point to common biological processes associated with it. However, when we analyzed the DEPs in each cell line, we found that a common biological process was associated with statistical significance to Tz resistance: metabolic pathways. Furthermore, we could establish that, in particular, lipid metabolism, the organophosphate biosynthetic process, and macromolecule methylation were pathways associated with Tz resistance in all three of our cell line models. We could not detect the enrichment of DEP in pathways strictly associated with cell proliferation. However, we acknowledge that those pathways we found to be modulated in our models may be indirectly linked to the enhanced proliferation rates detected in the SKBR-3 and MDA-MB-361 TzR cells. This may lessen the value of our study in understanding BCa resistance to Tz.

Lipid droplets are storage organelles pivotal for lipid and energy homeostasis. Altered lipid metabolism mediates drug resistance to small molecule inhibitors of HER2 kinase in breast cancer, raising a link between kinase signaling reprogramming and lipid metabolism [28]. Indeed, we observed significant changes in LD size or number within the three Tz-resistant cell lines by electron microscopy, pointing to a remodeling of their lipid production and or accumulation compared to wt. Our results are in line with previous reports suggesting that metabolic rewiring may contribute to Tz resistance. In particular, previous studies showed that fatty acid metabolism, membrane lipid rafts, and ERBB2 pathogenesis have close relationships [16] and that Tz increases lipogenesis via a FAS promoter [29]. Furthermore, a previous study showed that lapatinib-resistant cells undergo a CD63-mediated rewiring of the lipid metabolism [30]. Concerning the methylation of macromolecules, it is interesting to note that HSD17B4 methylation was identified as a predictive response marker to HER2-directed therapy in ERBB2+ BCa [31] and that genes involved in cancer-related pathways were frequently affected by epigenetic alterations in HER2-positive breast cancer [32]. Even more relevant is that epigenetic silencing of TGFBI was found to confer resistance to trastuzumab in human breast cancer [33]. Therefore, our results further underline the importance of methylation of macromolecules in the development of Tz resistance.

## 5. Conclusions

Our data strongly support the hypothesis that complex metabolic adaptation, including lipid metabolism, protein phosphorylation, and possibly chromatin remodeling, may fuel Tz resistance. Our study also identified a common set of 10 DEPs in all three TZ-resistant cell lines that may provide novel targets for therapeutic intervention.

## Figures and Tables

**Table 1:** Association of DEPs with pathways included in the KEGG collection and statistical significance as calculated by tools available at DAVID [https://david.ncifcrf.gov/tools.jsp], accessed on 17 October 2022. Shared pathways are in italic.

BT474 wt vs. Tz-R			MDA-MB-361 wt vs. Tz-R			SKBR-3 wt vs. Tz-R		
KEGG Pathway	% of DEPs	*p*-Value	KEGG Pathway	% of DEPs	*p*-Value	KEGG Pathway	% of DEPs	*p*-Value
*Metabolic pathways*	*18.6*	*5.00* × *10^−5^*	Chronic myeloid leukemia	2.9	1.40 × 10^−2^	*Metabolic pathways*	*28*	*7.00 × 10^−3^*
Biosynthesis of cofactors	4.3	5.00 × 10^−3^	TGF-beta signaling pathway	2.9	2.50 × 10^−2^	Thyroid hormone signaling pathway	6	1.10 × 10^−2^
			*Metabolic pathways*	*17*	*4.30* × *10^−2^*	Glycerophospholipid metabolism	5	2.40 × 10^−2^
			Proteoglycans in cancer	5	4.90 × 10^−2^			

**Table 2:** Top three clusters with their representative enriched identifier or terms [one per cluster]. Count is the number of DEPs with membership in the given reactome gene set identifier or gene ontology [GO] term. % is the percentage of all DEPs that are found in the given ontology term [only input proteins with at least one ontology term annotation are included in the calculation]. Log10[P] is the p-value in log base 10. Log10[q] is the multi-test adjusted *p*-value in log base 10. PATTERN shows the color code used for the protein lists where the term is found to be statistically significant, i.e., the multiple colors indicate a pathway/process that is shared across the three DEP lists [Red-SKBR-3; Blue-BT474; Green-MDA-MB-361]. The analysis was performed by tools available at https://metascape.org, accessed on 27 October 2022.

PATTERN	Identifier/Term	Category	Descrlptlon	Count	%	Log10(P)	Log10(q)
■ ■ ■	R-HSA-556833	Reactome Gene Sets	metabolism of lipids	36	8.98	−10.5	−6.15
■ ■ ■	GO:0090407	GO Biological Processes	organophosphate biosynthetic process	28	6.98	−8.89	−4.84
■ ■ ■	GO:0043414	GO Biological Processes	macromolecule methylation	11	7.97	−7.18	−3.13

## Data Availability

The mass spectrometry data have been deposited to the ProteomeXchange Consortium (https://www.ebi.ac.uk/pride/) with the dataset identifier PXD037657, accessed on 24 October 2022.

## Supplementary Materials

The following supporting information can be downloaded at: https://www.mdpi.com/article/10.3390/membranes13060540/s1, Figure S1: Box-and-whisker plot showing protein abundance levels in each cell line sample as determined by high resolution mass spectrometry analysis. Table S1: Correspondence between cell lines, trastuzumab resistance status, biological sample Id, technical replicates, and proteomic raw data files. Table S2: Results of the Brown-Forsythe and Welch ANOVA test with a Dunnett’s T3 multiple comparisons test of the ERBB2 expressed on the plasma membrane of the cell lines used in the study; Table S3: List of differentially expressed proteins (DEPS) in BT474, MDA-MB-361, and SKBR-3 Tz-R vs. wt cells. Table S4: Proteins relevant for resistance to ERBB2-targeted therapies in BCa found with high FDR confidence in samples from the cell lines used in the study
