# Supplementary figures and images for: Lipid Metabolism Reprogramming and Trastuzumab Resistance in Breast Cancer Cell Lines Overexpressing the ERBB2 Membrane Receptor
Source: Membranes (Basel). 2023 May 23;13(6):540. doi: 10.3390/membranes13060540 (PMC10304830; doi:10.3390/membranes13060540)

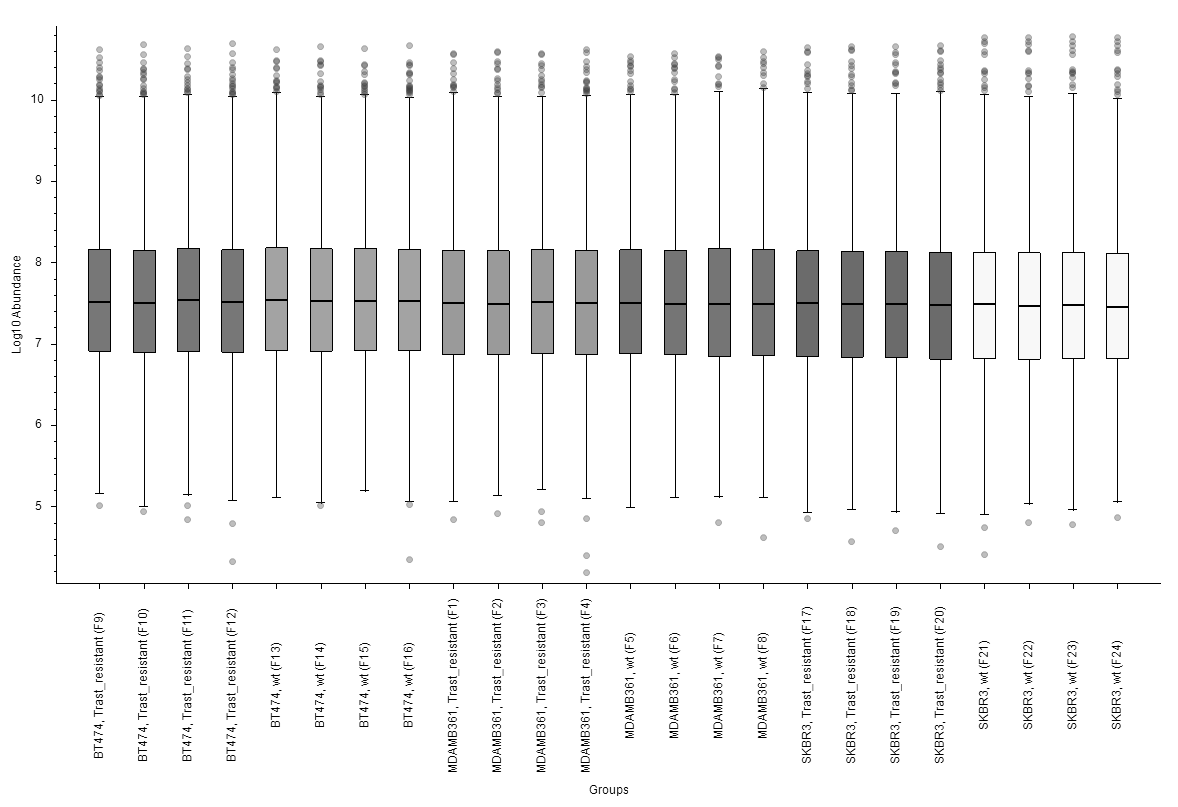

Supplement: Supplementary file 1 [file membranes-13-00540-s001.zip › Supplementary File 1.tiff]
